# Prevalence and sociodemographic factors associated with polysubstance use: analysis of a population-based survey in Jamaica

**DOI:** 10.1186/s12888-022-04160-2

**Published:** 2022-07-29

**Authors:** Kunal Lalwani, Patrice Whitehorne-Smith, Geoffrey Walcott, Joni-Gaye McLeary, Gabrielle Mitchell, Wendel Abel

**Affiliations:** 1 Dept of Community Health and Psychiatry, University of the West Indies, Mona, Kingston, Jamaica; 2 School of Public Health, Curtin University, Perth, Australia

**Keywords:** Polysubstance use, Jamaica, Household survey, Prevalence, Sociodemographic factors

## Abstract

**Background:**

In Latin America and the Caribbean, there is a dearth of research exploring polysubstance use. This study aims to determine the prevalence, varying combinations and associated sociodemographic characteristics of polysubstance use in Jamaica.

**Methods:**

This study involved a secondary data analysis of the Jamaica National Drug Prevalence Survey 2016 dataset where 4,623 participants between the age of 12 and 65 years from each household were randomly selected as respondents. Statistical analysis was performed to determine the prevalence and the sociodemographic correlates of polysubstance use among Jamaicans.

**Results:**

19.6% of respondents used two or more drugs in their lifetime. Of this amount 68.7% reported past year use and 61.9% reported past month use. Bivariate analyses reported polysubstance use was statistically significantly higher amongst males (U = 54,579, *p* = 0.000), those living in rural areas (U = 91,892, *p* = 0.003), non-Christian (U = 89,514, *p* = 0.014), and married persons (U = 74,672, *p* = 0.000). Past month polysubstance use was statistically significantly higher among employed persons than unemployed persons were (U = 81,342, *p* = 0.001). Surprisingly, there was a lack of significant differences between education level, household income and past month concurrent polysubstance use (*p* = 0.609; *p* = 0.115 respectively). Logistic regression model indicated males were 3.076 times more likely than females to report past month polysubstance use than females. Also, when compared to those 55–65 years old, participants 35–54 years were 2.922 times more likely and those 18–34 years were 4.914 times more likely to report past month polysubstance use. Additionally, those living in rural areas were 1.508 times more likely than participants living in urban areas to report past month polysubstance use. As it relates to occupational status, when compared to armed forces, skilled workers were 4.328 times more likely and unskilled workers were 7.146 times more likely to report past month polysubstance use.

**Conclusions:**

One in five Jamaicans identified as polysubstance users, predominated by marijuana as the most common factor amongst the polysubstance combinations examined, signalling the need for early marijuana interventions.

## Background

According to the latest World Drug Report, approximately 275 million people used drugs worldwide in the past year, and is expected to rise unprecedentedly to almost 300 million people by 2030 [1]. Drug use represents a massive public health issue facing the global community [2]. Latin America and the Caribbean are no exception, as more than 5.5 million persons are affected by drug use disorders [3].

Jamaica is the third largest island in the Caribbean and home to just under 3 million inhabitants [4]. Increasing drug use and illicit drug trade is a major problem in Jamaica [5]. Situated 145 km south of Cuba and 161 km southwest of Haiti [6], Jamaica’s location within the Caribbean Sea makes it an ideal transhipment point for drugs, especially from South America. As such, drug trafficking has led to the widespread availability of illicit drugs and given rise to increased domestic consumption, especially in a market where the cost for drugs is significantly below that seen internationally [7].

A multicultural society, the majority of Jamaicans practice Christianity [4] albeit with a growing Rastafarian movement that currently encompasses 29,000 followers [8]. Central to Rastafarian theology is the smoking of marijuana which is utilized as a religious sacrament [9, 10]. However, accompanying tobacco use as a means of “enhancing” the effects of marijuana have been recently documented amongst Rastafarians [11], and is a practice referred to as “boosting” [12] that at least one study has found to contribute to marijuana dependence [13]. Jamaica’s recent population survey indicated that marijuana and tobacco smoking were most prevalent (64.5% and 27.4% respectively) in this religious group, highlighting that targeted interventions are necessary in mitigating disproportionate burden of use amongst these individuals [14]. Furthermore, the frequent use of marijuana mixed with “grabba” or dried tobacco leaves [15] is not surprising, given that the two drugs are amongst the most frequently used by Jamaican youths [16].

Tobacco use is the leading cause of preventable deaths worldwide [17]. In the Caribbean, Jamaica has the second highest prevalence of current tobacco use [18], with smokers spending nearly 40% of their yearly income on tobacco-related products, and those with consequent lung disease spending more than 50% of their annual income treating their condition [19]. The alarming impact prompted Jamaica to sign onto the World Health Organization Framework Convention on Tobacco Control (WHOFTC), to combat the national epidemic of tobacco smoking.

Additionally, alcohol use is deeply embedded in Caribbean societies, rich in historical pastiche and cultural context, encompassing spiritual, social and medicinal roles [20, 21]. Yet excessive alcohol consumption poses a global health challenge [22–24]. According to the World Health Organization, Jamaica’s estimated alcohol per capita consumption rose from 4.9 L for both males and females in 2014 [25] to 11.9 L in 2018 [24], with over 40% of all Jamaicans engaging in heavy episodic drinking (HED), that is, consuming 60 or more grams of pure alcohol on at least one occasion monthly. In furtherance, a recent study examining alcohol consumption amongst Jamaicans during the COVID-19 pandemic, reported that approximately 45% and 49% of participants consumed more alcoholic beverages and used alcohol as a means of coping, throughout the pandemic respectively [26].

Notwithstanding, extant literature has predominantly focused on single-use substances, even though many people use more than one drug [27]. Polysubstance use can be defined as the use of more than one drug either concurrently or consecutively to amplify or neutralize another drug’s effect [28]. This study focused on concurrent use and may be defined as the use of two or more drugs within a given timeframe, spanning hours to days or even months [28–30].

Polysubstance use creates severe medical issues such as overdose, psychiatric co-morbidities such as depression, as well as more risky social behaviours such as promiscuous sexual practices, when compared to single drug users [31–37]. Furthermore, studies have concluded that the mortality rate is three times higher with polysubstance use versus singular drug use [38, 39].

Sociodemographic factors associated with polysubstance use include younger age [40–45], and male sex [46–50], although a growing body of research suggests a closing of the “gender gap” in overall drug patterns [51–53]. In addition, socioeconomic struggle with poverty, lack of housing and education predispose to heavier drinking and multiple drug use [54–57]. Numerous studies have found that being married and religiosity act as protective factors against drug use [52, 58–61]. Yet, there has been inconclusive evidence related to the role of employment status as worrisome substance use can result in unemployment [62], or alternatively, employment may perpetuate worrisome drug use and overindulgence due to increased means [63].

In continuing, studies have shown a higher prevalence of polysubstance use in persons with laborious, physical jobs in comparison to professionals and those employed in the armed forces [64–67]. Akin to employment status, much of the evidence regarding area of residence is debatable, as the literature highlights a diminishing gap between the patterns of substance use. Some studies concluded illicit substance use to be higher among rural individuals [68, 69], while others indicate a greater preponderance in urban areas [54, 70].

Globally, a number of studies have implicated the predominance of alcohol, tobacco and marijuana in polysubstance combinations [55, 56, 70–72]. Regionally, one study utilizing data collected from six countries in Latin America reported that the overall lifetime rate of polysubstance use is 21% [73]. In the Caribbean, a few studies have identified a similar predominant pattern of polysubstance use, albeit in smaller sample-sized studies. In Trinidad, Dhanookdhary and colleagues [74] examined substance use patterns amongst university students ranging from 17 to 50 years of age, and reported that 17% and 10% of the total sample indicated that they used a dual combination of alcohol and tobacco, and a triple combination of alcohol, tobacco, and marijuana in the past six-months respectively. In Jamaica, Harrison and colleagues [75] explored simultaneous polysubstance use among undergraduates at one university and reported that alcohol was the most frequent substance when combined with other drugs, followed by marijuana and tobacco. A more recent study utilized school surveys and examined current polysubstance use amongst adolescents in three Caribbean countries; namely Jamaica, Trinidad and Tobago, and the Dominican Republic, and found that 17.4%, 10.0% and 8.5% of participants engaged in polysubstance use respectively [76].

To date, no studies in the Caribbean have used nationally representative data to assess prevalence and the associated sociodemographic factors of polysubstance use. The relevant findings of this study may help provide impetus in prioritizing substance use treatment, especially in a country where almost half of all households live in rural areas [24]**.** Jamaicans living in rural areas who desire treatment are at a particular disadvantage as most services are concentrated in the urban areas [77], making access hard [78]. Furthermore, persons are usually treated in hospitals by physicians, rather than in a treatment facility with addiction specialists [79]. This can compromise the quality of care and perpetuate the issue of substance use.

The current study examined concurrent polysubstance use in a nationally representative sample of the Jamaican population to: 1) determine the prevalence of polysubstance use in Jamaica; 2) identify varying combinations of polysubstance use; and 3) elucidate the sociodemographic characteristics associated with polysubstance use.

## Methods

### Study design, participants and data source

The original study consisted of a cross-sectional survey conducted between April and July 2016 of a nationally representative sample of 4,623 participants between the age of 12 and 65 years [14]. The sampling design was developed by experts from the Statistical Institute of Jamaica (STATIN). Jamaica consists of 3 counties, 14 parishes and 5,771 Enumeration Districts (EDs), which are the smallest geographic units that allow for collection of survey data. Enumeration Districts (ED) were chosen in proportion to their numbers within the respective areas of residence. As a result, there were twenty-two (22) EDs per parish, with Sixteen (16) households per ED. The survey employed a stratified multi-stage cluster sampling design with EDs as the primary sampling units (PSU). The sampling interval for the selection of the dwelling from which the participant would be recruited was determined by dividing the total number of dwellings in the PSU by sixteen. A starting point was randomly used to determine the first dwelling from which a participant would be recruited. Using the Kish technique, one participant between the age of 12 and 65 years from each household was randomly selected as the respondent for the survey. Sampling weights were calculated by the probability of selection and non-response weights. Post-stratification weights based on parish level distributions of age and sex categories were applied to ensure that the distribution of the weighted sample matched the population distribution of both sex and age categories [14].

This study involved secondary data analysis of the population based National Drug Prevalence Survey 2016. The current study utilized the entire dataset population and extracted variables relevant to drug/polysubstance use patterns and sociodemographic factors. The sociodemographic data included age, gender, marital status, religious affiliation, geographical location, employment status, occupational description, household income and education. It followed demarcated guidelines that ensured the efficacy and applicability of the findings of retrospective studies [80]. Sequential steps were followed to minimize the limitations and strengthen the reliability of the data [81]. This involved outlining the research question and developing objectives followed by a systematic review of the literature pertinent to polysubstance use. Under established guidelines, a data extraction form was used to identify all the variables that were included in the current study design from the primary data set for analysis. This study contained no identifying data of respondents, and no direct or indirect contact was made with any respondents. There was no compensation in this secondary analysis.

### Measures

For this study concurrent polysubstance use was defined as the use of 2 or more of marijuana, tobacco and/or alcohol over a period of time. Lifetime prevalence was defined as any use during the person’s life, while past year prevalence was defined as any use during the previous year. Past month prevalence was examined as any use during the previous month.

### Data analysis

Descriptive statistical analysis was performed to determine the prevalence of concurrent polysubstance use as well as to describe the sociodemographic characteristics of participants and represented in frequencies, means and percentages. Concurrent polysubstance use was determined for the periods of lifetime or ‘ever’ use, past year use and past month use for alcohol, tobacco, and marijuana use. This was achieved by using the target variables related to “have you ever used…” for alcohol, tobacco and marijuana for lifetime, past year, and past month. “Yes” responses were coded as one and “No” were coded zero. Statistical computations were performed to score concurrent polysubstance use over each of the three periods. Respondents could only have a score of 0–3 for concurrent polysubstance use for each time. For analysis purposes, the use of 2–3 substances over lifetime, past year, and past month periods were considered concurrent polysubstance use. The limited range in the scoring system made it more prudent to represent concurrent polysubstance use variables as dummy variables. For inferential statistical analysis, past month concurrent polysubstance use, which represents current substance use was utilised as the dependent variable.

The Mann–Whitney U test and Kruskal–Wallis tests were performed to examine bivariate differences between subgroups of selected socio-demographic characteristics and past month concurrent polysubstance use. Binomial logistic regression model was generated to assess the likelihood of past month concurrent polysubstance use based on selected socio-demographic characteristics. The data were presented in the form of tables and text. A *p*-value of < 0.05 was considered statistically significant.

### Patient and public involvement

There was no involvement of patients or members of the public in conceptualisation, design, analysis, or interpretation of the results or write up of the paper.

### Ethical considerations

Ethical approval for the study was granted through the Ethics committee of The University of the West Indies, Mona Campus. There were no competing interests.

## Results

There were 4623 respondents in the study of which 54.8% (*n* = 2535) were females and 45.2% (*n* = 2088) were males. Table 1 shows the socio-demographic characteristic of study respondents. The age range of respondents was between 12–65 years with a mean age of 36.18 years (s.d. ± 14.815). Most respondents lived in rural areas (58.6%), were married (68.7%), Christian (71.0%), unemployed (52.9%), skilled workers (72.1%) and had less than a university level education (82%).

**Table 1:** Sociodemographic characteristics of study respondents

Variable	*f*	%
*Gender*		
Male	2088	45.2
Female	2535	54.8
*Age group*		
12–17 years	544	11.8
18-34 years	1708	36.9
35-54 years	1712	37.0
55-65 years	659	14.3
*Geographical Location*		
Urban	1914	41.4
Rural	2709	58.6
*Marital Status*		
Married/Common law	3167	68.7
Unmarried	1446	31.3
*Religious beliefs*		
Christian	3265	71.0
Non-Christian	1332	29.0
*Education level*		
primary or less	584	12.7
secondary school	3197	69.3
university	832	18.0
*Employment status*		
Employed	2177	47.1
Unemployed	2446	52.9
*Occupational status*		
Professional	248	11.3
Skilled workers	1584	72.1
Unskilled workers	328	14.9
Armed forces	37	1.7
*Total household income*		
less than $25,000	1040	26.6
$25,001-$50,000	1337	34.2
$50,001-$70,000	719	18.4
$70,001- $100,000	455	11.6
$120,000 or more	359	9.2

### Substance use

Table 2 demonstrates the prevalence of use of alcohol, tobacco, and marijuana as well as concurrent polysubstance use. Most respondents reported having used alcohol in their lifetime (74.8%). Meanwhile a similar number of respondents reported lifetime use of tobacco and marijuana (28.1% and 28.3% respectively). Among those who reported lifetime use of alcohol, 72.9% reported use in the past year and 54.3% reported use in the past month. Of these three substances the lowest past year and past month use was seen for tobacco. Approximately 20% (*n* = 907) of respondents reported lifetime ‘ever use’ concurrent polysubstance use and past year and past month use was represented at 68.7% and 61.9% respectively.

**Table 2:** Substance use prevalence and combination for concurrent polysubstance use

Substance Use	Lifetime “Ever Use”	Past Year	Past Month
Alcohol	3460 (74.8%)	2522 (72.9%)	1879 (54.3%)
Tobacco	1299 (28.1%)	561 (43.2%)	494 (38%)
Marijuana	1307 (28.3%)	786 (60.1%)	704 (53.9%)
*Concurrent Polysubstance Use* (use of 2 or more drugs)	907 (19.6%)	623 (68.7%)	561 (61.9%)
*Combination Polysubstance Use*			
alcohol + tobacco		504 (10.9%)	421 (9.1%)
alcohol + marijuana		598 (12.9%)	710 (15.4%)
tobacco + marijuana		406 (8.8%)	355 (7.7%)
alcohol + tobacco + marijuana		377 (8.2%)	318 (6.9%)

Table 2 also illustrates the combination of substances used among respondents. The most common combination of two or more substances over the past year and past month was those who reported using both alcohol and marijuana without tobacco (12.9% and 15.4% respectively). Those who used both alcohol and tobacco (without marijuana) at 10.9% for past year and 9.1% for past month followed this. The least common combination was for respondents who used alcohol, tobacco and marijuana in the past year or past month at 8.2% and 6.9%.

For bivariate analysis past month polysubstance use was used as the dependent variable (*n* = 907). A test of Skewness and Kurtosis on the target variable for past month polysubstance use revealed skewness of -0.0.489 and kurtosis of -1.765 indicating that the data is slightly skewed. Consequently, non-parametric statistics were used to analyse the data to produce the most accurate results. Mann–Whitney U tests were performed to analyse the difference between the dichotomous independent variables related to sociodemographic factors and past month concurrent polysubstance use. The data indicates that past month polysubstance use was statistically significantly higher among males than females (U = 54,579, *p* = 0.000), those who lived in rural areas than urban (U = 91,892, *p* = 0.003), non-Christian than Christian (U = 89,514, *p* = 0.014), married than unmarried (U = 74,672, *p* = 0.000) and employed than unemployed (U = 81,342, *p* = 0.001) (Table 3).

**Table 3:** Mann–Whitney U test of selected sociodemographic factors and past month concurrent polysubstance use

Sociodemographic factors	N	Mean Rank	Sum Rank	U	*p* value
*Gender*					
Male	685	485.32	332,446	54,579	0.000*
Female	222	357.35	79,332		
*Geographical Location*					
Urban	407	429.78	174,920	91,892	0.003*
Rural	500	473.72	236,859		
*Religious beliefs*					
Christian	534	435.13	232,359	89,514	0.014*
Non-Christian	365	471.76	172,191		
*Marital Status*					
Married/Common law	632	471.35	297,892	74,672	0.000*
Unmarried	273	410.52	112,073		
*Employment status*					
Employed	604	470.83	284,380	81,342	0.001*
Unemployed	303	420.46	127,398		

^*^ *p* < 0.01 level significance

Additionally, Table 4 shows results from Kruskal–Wallis tests which indicated significant differences between age group, occupation status and past month concurrent polysubstance use (χ2 = 33.479, *p* = 0.000; χ2 = 35.262, *p* = 0.000 respectively). As it relates to age group and past month concurrent polysubstance use, Dunn’s pairwise tests carried out (adjusted using the Bonferroni correction) for the four pairs of groups found that the age group 35–54 years had a statistically significant difference in mean rank than those in the age group 55–65 years (mean ranks = 461.25; 359.31 respectively, *p* = 0.000) for past month polysubstance use. A statistically significant difference was also found between the means for respondents 18–35 years and those 55–65 years (mean ranks = 482.34; 359.31 respectively, *p* = 0.000).

**Table 4:** Kruskal–Wallis test of selected sociodemographic factors and past month concurrent polysubstance use

Sociodemographic factors	N	Mean Rank	df	χ2	*p* value
*Age group*					
12–17 years	26	452.58			
18-34 years	395	482.34	3	33.479	0.000*
35-54 years	342	461.25			
55-65 years	144	359.31			
*Education level*					
Primary or less	119	454.89			
Secondary school	653	456.23	2	0.993	0.609
University	133	435.47			
*Occupational status*					
Professional	31	161.52			
Skilled workers	437	290.18	3	35.262	0.000*
Unskilled workers	87	309.31			
Armed forces	12	192.00			
*Total household income*					
less than $25,000	218	389.49			
$25,001-$50,000	262	378.23			
$50,001-$70,000	139	383.61	4	7.435	0.115
$70,001- $100,000	76	345.80			
$120,000 or more	55	327.59			

^*^ *p* < 0.01 level significance

There was no other statistical significance noted between other age groups and past month concurrent polysubstance use.

As for occupation status and past month concurrent polysubstance use, Dunn’s pairwise tests (adjusted using the Bonferroni correction) found statistically significant differences between three groups. Skilled workers had significantly higher means than professionals did for past month polysubstance use (mean rank = 290.18; 161.52, respectively, *p* = 0.000). Unskilled workers also had significantly higher means than professionals did for past month polysubstance use (mean rank = 309.31; 161.52, respectively, *p* = 0.000) as well as those respondents working in the armed forces (mean rank = 309.31; 192.00, respectively, *p* = 0.000).

There was no other statistical significance noted between other occupation groups and past month concurrent polysubstance use. Table 4 also illustrates that no significant differences were found between education level, household income and past month concurrent polysubstance use (*p* > 0.05 respectively).

Table 5 shows the results of a binomial logistic regression analysis performed to assess associations between concurrent past month polysubstance use and sociodemographic factors of gender, age, geographical location, marital status, religious beliefs, and occupation status. The categories for age were modified to exclude those 12–17 years as this represented a singular case and outlier in the analysis.

**Table 5:** Logistic regression of selected sociodemographic factors and past month concurrent polysubstance use

Sociodemographic factors		95% C.I	
	Odds Ratio	Lower	Upper
*Gender*			
Male	3.076	1.874	5.051
Female	reference		
*Age*			
18–34 years	4.914	2.779	8.686
35–54 years	2.922	1.742	4.903
55–65 years	reference		
*Geographical location*			
Rural	1.508	1.002	2.270
Urban	reference	0.942	2.262
*Marital status*			
Unmarried	1.346	0.896	2.022
Married	reference	0.442	1.050
*Religious beliefs*			
Non-Christian	1.347	0.898	2.019
Christian	reference		
*Occupation status*			
Professional	0.677	0.145	3.168
Skilled workers	4.328	1.191	15.718
Unskilled workers	7.146	1.792	28.501
Armed forces	reference		

The model illustrated that males were 3.076 times more likely than females to report past month polysubstance use than females. Also, when compared to those 55–65 years old, participants 35–54 years were 2.922 times more likely, and those 18–34 years were 4.914 times more likely to report past month polysubstance use. Additionally, those living in rural areas were 1.508 times more likely than participants living in urban areas to report past month polysubstance use. As it relates to occupational status, skilled workers were 4.328 times more likely and unskilled workers were 7.146 times more likely to report past month polysubstance use. This model indicated no significant associations between marital status and religious status and past month polysubstance use.

## Discussion

This is the first study in Jamaica and the Caribbean that demonstrates the prevalence of concurrent polysubstance use, combinations of substances used, and associations with sociodemographic factors. This research found that approximately 1 in every 5 Jamaicans aged 12–65 years used two or more drugs in their lifetime. This is comparable to our Latin American neighbours that undertook a similar survey and reported that 21% of participants combined at least two substances [73]. However, inconsistent with other international findings that report alcohol and tobacco as the most prevalent combination, this study highlighted alcohol and marijuana as the predominant combination in the Jamaican population [82, 83].

Although alcohol, marijuana and tobacco were the most common three-substance combination in Latin America, the prevalence ranged from 0.1% to 1.9% for all six countries [73]. In Jamaica, the current use of alcohol, marijuana, and tobacco combination was 6.9%. This represents a more than three times increase relative to our regional neighbours. Our study indicates that marijuana plays a primary role in the combinations reported. The strong predilection towards marijuana use is likely multi-factorial, reflective of recent decriminalization legislation that one study indicated had a positive association with the use of marijuana [84], and strong socio-cultural validation and Rastafarian ideology and influence [9, 84–86]. Notwithstanding, these findings are indicative of a polysubstance pattern that is noteworthy and contrasting to our regional and international counterparts. This represents a unique challenge to stakeholders that requires further research to elucidate appropriate reduction strategies.

As it relates to socio-demographic factors correlated with concurrent polysubstance use, bivariate analysis revealed that gender, age, geographical location, religious beliefs, marital status, employment status and occupational status were associated factors. The multivariate logistic regression analysis indicated that being an unskilled worker, younger age, male gender and living in rural areas were predictive of concurrent polysubstance use.

Unskilled workers were approximately seven times more likely and skilled workers were four times more likely to report past month concurrent polysubstance use than the persons working in the armed forces. This is in keeping with the literature that found skilled and unskilled labourers had the highest figures of drug and alcohol use [64–66]. In addition, persons in the armed forces were less likely to engage in past month concurrent polysubstance use as the Jamaica Defence Force and Jamaica Constabulary Force (JCF) have instituted random drug testing since 2000 [87]. In Jamaica, high levels of substance use amongst unskilled workers is a major concern, as most unskilled labourers are daily wagers or are paid on a weekly basis. Furthermore, informal sector workers tend to be compensated on a lower pay scale than their counterparts [88, 89]. Hence, if these workers have a substance use habit, it will reduce a major share of their income and could prove a hindrance to economic sustenance.

Additionally, respondents between the ages of 18–34 and 35–54 years were approximately five and three times more likely, than those in the older age group 55–65 years respectively, to report past month concurrent polysubstance use. These findings are comparable to global literature that suggests young adults as being the predominant polysubstance users [37, 56, 72, 90], with most of the problematic drug users being in their 20’s [91], and with use becoming less frequent as persons become older [92]. This may be linked to a greater willingness among young people to experiment and use drugs to enhance social interactions [93].

Moreover, gender was also found to be predictive of concurrent polysubstance use with females being three times less likely to report use than males [48–50, 73, 90]. This underscores the need for crafting and implementing gender-based strategies in dealing with the issue of polysubstance use in the general population.

Past month polysubstance use was also higher amongst those who lived in rural than urban areas, with those living in rural areas being approximately one and a half times more likely to report use. This is in keeping with other research that suggests drug use including polysubstance use, have increased in the rural environment, and have in fact outpaced their urban counterparts [68, 69]. This finding is likely as a result of population distribution where almost half of all Jamaican households live in rural habitats [24]. However, to the detriment of Jamaicans living in rurality and desirous of treatment, most services are concentrated in the urban areas [77] and this represents a barrier to access [78]. Lack of access and available options means that persons tend to have to pay more for substance abuse treatment and may not be able to afford it [79, 94, 95]. Also, rural life does not lend to anonymity and as such, the social stigma associated with having a drug problem and/or requiring treatment for same, makes seeking treatment difficult, especially if they know the professional offering them treatment [79, 95, 96].

Although, not reflected in the logistic regression model, bivariate analysis indicated associations between marital status, religious beliefs, and employment status and past month concurrent polysubstance use. Concurrent polysubstance use was higher among the married than unmarried population. This contrasts with the literature that posits being married as a protective factor associated with better drug abuse outcomes [52, 97, 98]. One possible explanation is that the survey limited questions regarding marriage to marital status rather than the nature of the marital relationship between individuals, that the literature suggests may predict lower rates of drug use [98]. Notwithstanding, at a societal level, divorce still carries a stigma while conversely, marriage represents a status marker for both Jamaican men and women that is associated with a level of societal prestige that is otherwise unattainable [99]. These findings suggest the need for further research to examine the role of substance use amongst married persons, and whether it serves as a means of coping with possible marital discord.

Additionally, past month polysubstance use was higher among non-Christian than Christian persons. This finding is in keeping with what has been reported previously, endorsing the protective role of religious affiliation [58, 60, 61]. In our study, the non-Christian population would have included persons who prescribed to other religious affiliations like Rastafarianism and/or were non-religious. The island’s most recent survey highlighted marijuana use as being most prevalent amongst Rastafarians [14]. Further local studies may provide evidence of the impact of marijuana as a possible gateway drug [100] correlated with developing other drug use in these individuals. This may prove insightful in generating a local public health response, targeting church-based and other local religious populations.

As it relates to employment, past month polysubstance use was higher among employed than unemployed persons. This is in keeping with more recent studies, which note higher polysubstance use in individuals who are employed [101, 102].

Notably, there was a lack of significant differences between education level, household income and past month concurrent polysubstance use (*p* > 0.05). These findings are contrary to the literature that reports a strong association between socioeconomic struggle and polysubstance use, particularly amongst persons with a lower household income and having less than a high school education [55, 56]. This finding is likely due to the socio-cultural validation of alcohol and marijuana use in Jamaica and the Caribbean at large [20, 21, 85, 103]. Pre-existent literature highlights that society’s approval of drug use is influential and permissive of use [43, 104, 105].

## Conclusions

The present study suggests widespread polysubstance use amongst the Jamaican population, predominated by the presence of marijuana as the most common factor within the combinations examined, and signal the need for early marijuana interventions.

The sociodemographic associations seen amongst polysubstance users suggest the need for a more inclusive approach by not just limiting the focus to single-use substances, but rather further widening the scope of enquiry, assessment, treatment, and rehabilitative efforts, to include and subsume polysubstance use in the restructuring and reformation of drug policies and programmes.

### Strengths and limitations of this study

The is the first study in Jamaica to examine the issue of polysubstance use from a nationally representative sample, by eliciting a sociodemographic profile of individuals engaging in polysubstance use and determining prevalence rates. The strength of the secondary analysis conducted was augmented by the utilization of the entire dataset population. This study was a cross-sectional design and thus is limited to creating association. This study utilised a self-report survey related to substance use behaviour and thus findings may suffer from underreporting bias. Additionally, the household survey questionnaire focused mostly on single substance use disorders and lacked specific questions related to polysubstance use. Moreover, the measure for concurrent polysubstance use may have been limited in its scope, necessitating a broad definition of the term to be utilised in this study to provide baseline extrapolation about its use in Jamaica. This approach provides a foundation for future research.

### Recommendations and future research

The term polysubstance use, inclusive of its definition, social and health impact, has not been included in the messaging by the relevant bodies, as part of addressing substance use education on a national scale. Dissemination of information as part of a prevention strategy could help to improve awareness and knowledge regarding polysubstance use, and contribute to mitigating its impact at a societal level.

Future epidemiological research can incorporate measures of frequency and severity to expand the measures of substance use in further establishing the profile of the polysubstance user. Consideration to conducting a cohort study, examining patterns of polysubstance use longitudinally as individuals’ transition from adolescence to young adulthood, and subsequently older adulthood, may prove invaluable.

The initial national household survey was conducted shortly after the amendment to the Dangerous Drugs Act in Jamaica, which allowed for new provisions regarding the possession and smoking of marijuana, its use for therapeutic/scientific purposes and its use by persons of the Rastafarian faith. As such, further studies can be done to ascertain whether marijuana decriminalization has extended influence towards polysubstance use, given that a worthwhile period would have elapsed since the initial study.

## Data Availability

The data that support the findings of this study are available from the National Council on Drug Abuse, Jamaica, and the Inter-American Drug Abuse Control Commission (CICAD) but restrictions apply to the availability of these data, which were used under license for the current study, and so are not publicly available. Data are however available from the authors upon reasonable request and with permission of the National Council on Drug Abuse, Jamaica, and the Inter-American Drug Abuse Control Commission (CICAD). For access to the database, contact Mrs. Uki Atkinson, research analyst, at uatkinson@ncda.org.jm.
